# Risk factors and patterns of onset in binge eating disorder

**DOI:** 10.1002/eat.20208

**Published:** 2005-10-17

**Authors:** Jamie L Manwaring, Anja Hilbert, Denise E Wilfley, Kathleen M Pike, Christopher G Fairburn, Faith-Anne Dohm, Ruth H Striegel-Moore

**Affiliations:** 1 Department of Psychology, Washington UniversitySt. Louis, Missouri; 2 Department of Psychiatry, Washington UniversitySt. Louis, Missouri; 3 Department of Medicine, Washington UniversitySt. Louis, Missouri; 4 Department of Pediatrics, Washington UniversitySt. Louis, Missouri; 5 Department of Psychology, Washington UniversitySt. Louis, Missouri; 6 Department of Psychiatry, Columbia UniversityNew York, New York; 7 Department of Psychiatry, Oxford UniversityOxford, England; 8 Graduate School of Education and Allied Professions, Fairfield UniversityFairfield, Connecticut; 9 Department of Psychology, Wesleyan UniversityMiddletown, Connecticut

**Keywords:** risk factors, otiology, binge eating disorder, psychopathology, binge-first, diet-first

## Abstract

**Objective:**

The current study examined risk factors in women with binge eating disorder (BED) who began binging before dieting (binge-first [BF]) compared with women with BED who began dieting before binging (diet-first [DF]). It further aimed to replicate findings regarding eating disorder and general psychopathology among BF versus DF subtypes.

**Method:**

One hundred fifty-five women with BED completed the Oxford Risk Factor Interview to retrospectively assess risk factors occurring before eating disturbance onset. Clinical interview assessed eating disorder and general psychopathology.

**Results:**

Overall, no significant differences in risk factors emerged between the groups. The BF group had a significantly earlier onset of BED than the DF group. In contradistinction to previous studies, the DF group endorsed more eating disorder psychopathology and lifetime diagnosis of any substance use disorder.

**Conclusion:**

Limited support was seen for different risk factors in BF versus DF women, suggesting similar etiologic pathways in both subtypes. © 2005 by Wiley Periodicals, Inc.

## Introduction

Binge eating disorder (BED) is included in the 4th ed. of the Diagnostic and Statistical Manual of Mental Disorders (DSM-IV) as a provisional eating disorder diagnosis in need of further study.^1^ Although its inclusion has generated an impressive body of research, the etiology of BED is still relatively unclear and made more uncertain by the heterogeneity within the BED population. This hetero-geneity is demonstrated by the large male and ethnically diverse patient profile of BED, as well as diverse symptomatology such as the differences seen in the clinical presentation of Black and White women with BED.^2^–^5^

To further delineate and understand the heterogeneity of this disorder, researchers have attempted to subtype BED, predominantly by distinguishing individuals whose binge eating precedes their dieting (binge-first [BF]) from those whose dieting precedes their binge eating (diet-first [DF]). Subtyping has per-meated the literature across psychiatric disorders, from schizophrenia to posttraumatic stress disorder,^6^,^7^ and the eating disorder field contains formal diagnostic subtypes for anorexia nervosa (AN; restricting or binge eating/purging subtype) and buli-mia nervosa (BN; purging or nonpurging subtype).^1^ Establishing subtypes for psychiatric disorders such as BED depends partially on the extent to which individuals within various subtypes consistently differ on significant variables such as symptom presentation and treatment response (see Stice et al.^8^).

Research on the BF versus DF subtypes has found that binge eating before dieting occurs in approximately 35%–55% of BED patient samples, with the variability likely due to researchers’ different approaches to conceptualizing and assessing ‘‘dieting’’ (e.g., using a set weight loss as a criterion for significant dieting vs. using a participant’s definition of dieting).^9^–^13^ When examining differences between the groups, the BF group has been shown to report the following: binge eating at an earlier age (e.g., 11– 13 years for BF vs. 25–26 years for DF); a higher frequency of weight-related teasing; an earlier onset of overweight and BED diagnosis; a higher number of lifetime psychiatric diagnoses and history of substance use disorders; and a greater likelihood of having an Axis II personality disorder.^9^,^10^,^13^ The only exception to the BF group being more symptomatic was a finding from one study that found the DF group to be more likely to receive a lifetime diagnosis of stimulant abuse.^9^ In addition, it is possible that the BF versus DF distinction has predictive validity. For example, being in the BF group or having an early age of onset of binge eating (at or before age 16) has been found to be related to poor treatment outcome.^14^–^16^

The evidence supporting the distinction between the clinical presentation of BF and DF subtypes raises the possibility that different etiologic pathways may lead to BED. It was originally believed, due to similarities in symptomatology with BN, that the etiology of BED would conform to a model initially proposed for BN that emphasizes restraint. In this restraint model, dietary restriction is exercised by adopting a cognitively regulated eating style, causing susceptibility to disinhibition and consequent binge eating. The model would then posit that BED subjects are primarily of the DF group. However, it may not be suited to describe the BF subtype of BED.^17^,^18^ Although the role of dietary restraint in the etiology and maintenance of BED remains unclear,^19^–^22^ other factors such as affect regulation and family conflict have been implicated in the initiation and maintenance of binge eating in BED (e.g., Fairburn et al.^23^ and Striegel-Moore et al.^24^), supporting models that instead propose a more pronounced etiologic role for inter-personal and psychological factors.^25^ With respect to risk factors, developmental differences between the BF and DF subtypes remain largely unexplored.

Because previous studies on the BF versus DF subtyping of BED predominantly investigated Caucasian, treatment-seeking samples and lacked valid criteria for determining the onset of dieting and/or binge eating (e.g., using a set weight loss as a criterion for significant dieting), the current study aims to replicate previous findings on a characteristic psychopathologic profile of BF versus DF by (a) using a community-based sample of Black and White women with BED and (b) utilizing an interview format with a stringent definition of dieting, likely increasing the reliability and validity of BF versus DF subtyping. A further main purpose of this study is to investigate risk factors in women with BF and DF subtypes of BED. These aims would also assist in elucidating the construct validity of subtyping BED through the BF versus DF distinction.^26^ It is expected that, consistent with earlier reports, the BED sample will be divided approximately equally between the BF and DF groups, with the BF group having begun binge eating and diagnosed with BED at an earlier age than the DF group. The BF group is also hypothe-sized to endorse greater lifetime psychopathology and report greater exposure to risk factors than the DF group, consistent with previous literature.

## Method

### Recruitment

The New England Women’s Health Project (NEWHP) recruited women from the community via advertisements for a study on women’s mental health, as well as from respondents to a telephone survey of randomly selected households. (For recruitment details see Striegel-Moore et al.^5^). The women had to be either Black or White, born in the United States, be between the ages of 18 and 40 years, and living within driving distance of the nearest project office. Women were excluded if they did not meet these criteria, had a physical condition known to influence eating or weight, were currently pregnant, or had a current psychotic disorder. To determine BED status, participants were first interviewed using a telephone-screening interview, and then invited to an in-person interview if deemed eligible from the telephone screen.

### Participants

#### Sample

The original sample consisted of 162 women, which was then reduced to a sample of 155 women due to exclusion criteria for the purpose of the current study: Seven participants were excluded because they either reported the same age of onset for both dieting and binge eating, or they reported engaging in compensatory behaviors other than dieting before the onset of binge eating. The final sample comprised 155 women with a current diagnosis of BED (Black women: n = 59 [38% of the sample]; White women: n = 96 [62% of the sample]) with a current mean age of 31.17 years (SD = 5.73) and a mean body mass index (BMI) of 34.66 kg/m^2^ (SD = 10.14). A significant difference was found between the two races in terms of BMI, with Black women having a significantly higher BMI than White women (mean BMI = 36.80 kg/m^2^, SD = 9.36 vs. mean BMI = 33.36 kg/m^2^, SD = 10.41; t = 3.69, df = 152, p < .05). Because no significant demographic differences (i.e., age, level of education, marital status) were seen between the Black and White women, and a chi-square analysis revealed that the racial distribution did not significantly differ in the BF versus the DF group (χ^2^_1_ = 3.24, p > .05), further analyses were based on a combined Black and White sample.

Fifty-two percent of the sample was single or never married, 34% were married or living with partner, 10% were separated or divorced, and 1% was widowed (marital status not available for 3% of sample; marital status did not significantly differ in the BF vs. DF group, *p* = .092). Nineteen percent of the sample completed high school, 41% completed some college, 23% were college graduates, 13% completed some graduate or professional school, and 2% had a graduate or professional degree (educational level not available on 2% of the sample).

### Primary Assessments

#### Eating Disorder Examination (EDE)

The key diagnostic features of eating disorders (e.g., number of binge eating days and episodes, number of purging episodes, importance of shape or weight) were assessed with an abbreviated, diagnostic version of the EDE^a^. The EDE is a standardized, investigator-based interview with established reliability and validity.^27^,^28^ Assessors for this sample received extensive training and ongoing super-vision in the administration of the interview. The EDE was used in the current study to assess participants’ clinically significant eating disturbances and to define a participant as having a BF or DF onset.

Concerning the BF/DF classification, onset of eating disturbances was conservatively defined as the age at which the first significant and persistent behaviors characteristic of an eating disorder began, that is, recurrent overeating, sustained dieting, and/or purging.^29^ This ‘‘index age’’ was then utilized as a marker throughout the interview in determining events and behaviors occurring before this age. Dieting was defined as attempts to eat less than most people (using <1200 kcal/day as a guide) for at least 3 months. Recurrent over-eating was defined as having an OBE (i.e., eating what would generally be considered an unusually large amount of food, accompanied by a sense of loss of control^27^) at least once a week for 3 months. Participants were classified as having a BF onset if their index age for recurrent overeating occurred before their index age for sustained dieting, whereas a DF classification was made for those participants whose index age for sustained dieting occurred before their index age for recurrent overeating. Participants who had never engaged in sustained dieting were determined to have a BF onset.

#### Oxford Risk Factor Interview (RFI)

The current study used a modified version of the RFI, a semistructured, investigator-based interview used to measure factors purported to place an individual at risk for an eating disorder.^23^ Previous studies, using similar vigorous training and supervision procedures, have established the interrater reliability of the RFI across the risk factors (mean weighted kappa = 0.66, SD = 0.17).^29^ The interview was used in this study to assess risk factors for, and antecedents of, participants’ clinically significant eating disturbances.

For the assessment of risk factors, the interview used behavioral definitions of key concepts to minimize problems associated with retrospective reporting.^30^ Items had to be rated on ordinal scales based on severity or frequency to assess degree of exposure to a potential risk factor (from a null score indicating no exposure to a score of 3 or 4 indicating severe or frequent exposure). The risk factor items measured by the RFI were categorized a priori into six broad conceptually related risk domains (Subject’s Mental Health, Subject’s Physical Health, Other Environmental Experiences, Family Weight and Eating Concerns, Quality of Parenting, and Parental Psychopathology). The items in each risk domain were then factor analyzed using principal components factor analysis with varimax rotation (each risk domain was factor analyzed separately). The factor analyses resulted in 21 risk subdomains, each reflecting certain types of exposure^b^. (See Striegel-Moore et al.^24^)

The RFI was also used to assess potential antecedents of the eating disorder. These potential antecedents included life event stressors occurring the year before the index age (e.g., moves, illnesses, or the ending of a significant relationship).

### Additional Measures

#### Eating Disorder Examination-Questionnaire (EDE-Q)

Eating disorder psychopathology was assessed by the EDE-Q, the self-report form of the EDE, which includes the subscales of Restraint, Eating Concern, Weight Concern, and Shape Concern.^31^,^32^

#### Structured Clinical Interview for DSM-IV (SCID-IV)

Life-time Axis I psychiatric disorders were assessed using the SCID-IV, a standardized, investigator-based interview with established reliability and validity.^33^,^34^

#### Parental Bonding Instrument (PBI)

The PBI, a questionnaire with established reliability and validity, assessed participants’ experience of both parents during their first 16 years of life, from which were extracted measures of parental affectionless control, overprotection, and low care.^35^,^36^

#### BMI

Height and weight were obtained to calculate BMI (in kg/m^2^).

### Data Analysis

Groups were compared using chi-square analyses for categorical variables (e.g., from SCID-IV, RFI, and PBI). SCID-IV diagnoses with cell frequencies below five were recoded into diagnostic categories (e.g., all anxiety dis-orders were collapsed into the diagnostic category anxiety disorders). For continuous variables from the EDE and EDE-Q, groups were compared using independent-sample t tests, accounting for heterogeneity of variances. Effect sizes were reported when appropriate for t tests (Cohen’s d: small = .20, medium = .50, and large = .80), as well as for chi-square tests (Φ for *df* = 1: small = .10, medium = .30, and large = .50).^37^ Given the number of comparisons examined, an alpha level of *p* < .01 was applied to all statistical analyses.

This research was reviewed and approved by an institutional review board.

## Results

### Demographic and Clinical Description of BF Versus DF Groups

Of the 155 women in our sample, 125 women (81%) began binge eating before dieting, whereas 30 women (19%) began dieting before binge eating (Table 1). Sixty-eight (54%) women in the BF group had never engaged in sustained dieting, whereas the remaining 57 (46%) women in the BF group began sustained dieting after the onset of consistent binge eating. The overall mean index age of the sample was 15.72 (*SD* = 7.21). As expected, a significant difference was found between the two groups in the age of onset of regular binge eating, with the BF group being significantly younger (mean age of regular binge eating = 19.07, *SD* = 8.48) than the DF group (mean age of regular binge eating = 24.52, *SD* = 7.33; *p* = .002, *d* = .66). Also as expected, the BF group had a significantly earlier age of onset of BED (mean age of onset = 20.71, *SD* = 8.48) than the DF group (mean age of onset = 25.37, *SD* = 7.51; *p* = .007, *d* = .56).

**Table 1:** Sample characteristics of binge-first (BF) and diet-first (DF) groups

	*Binge-First n* = 125		*Diet-First n* = 30					
	M	SD	M	SD	t	df	p(t)	d
Age (in years)	2	30.70	5.83	32.07	5.46	−1.16	153	.246
Body mass index (in kg/m )	34.92	10.59	33.04	8.05	0.91	150	.366	.19
Age of onset of regular BE (3 mths)	19.07	8.48	24.52	7.33	−3.19	150	.002	.66
Age of onset of BED (in years)	20.71	8.48	25.37	7.51	−2.76	153	.007	.56
Age of onset of dieting^a^	(in years)	20.23	6.40	17.87	6.12	1.66	85	.100

a Based on a BF sample that had sustained dieting (n ¼ 57).

With respect to eating disorder and general psy-chopathology, comparisons indicated that the DF group endorsed significantly higher Restraint and Weight Concern than the BF group, as assessed by the EDE-Q (*p* < .01, *d* ≥ .51), with a trend towards significantly higher Eating Concern, Shape Concern, and ‘‘Importance of Weight or Shape’’ for the past 6 months, as assessed by the EDE (*p* < .05, *d* ≥ .43) (Table 2). There were no significant differences between the groups for summed frequencies of life-time Axis I disorder, mood disorder, or anxiety dis-order as assessed by the SCID-IV (*ps* > .01). However, women in the DF group were significantly more likely to be diagnosed with a lifetime substance use disorder than were women in the BF group (*p* = .002, Φ = .26), likely because of a trend towards a significantly greater history of alcohol abuse or dependence than the BF group (χ^2^_2_ = 8.25, *p* = .016) (Table 3).

**Table 2:** Comparison of eating symptomatology between binge-first (BF) and diet-first (DF) groups

	*Binge-First n* = 125		*Binge-First n* = 125				
	*M*	*SD*	*M*	*SD*	*t*	*p(t)*	*d*
Eating disorder psychopathology							
EDE-Q							
Restraint	1.93	1.41	2.71	1.28	−2.77^d^	.006	.57
Eating Concern	2.67	1.32	3.26	1.08	−2.27^d^	.025	.47
Weight Concern	3.79	1.39	4.45	0.92	−3.09^d^	.003	.51
Shape Concern	4.16	1.39	4.73	0.99	−2.11^d^	.037	.43
EDE							
Importance of Weight or Shape^a^	2.98	0.78	3.30	0.60	−2.09^d^	.039	.43
Disordered eating behavior							
EDE							
Objective bulimic episodes^b^	18.81	13.85	15.87	8.02	1.12^e^	.266	.23
Subjective bulimic episodes^b^	5.24	10.95	9.67	14.51	−1.83^d^	.069	.38
Purging^c^	0.18	0.50	0.17	0.46	0.17^e^	.862	.04

Note: EDE = Eating Disorder Examination; EDE-Q = Eating Disorder Examination-Questionnaire.

a On a scale from 1 (*no importance*) to 4 (*most important*), importance of shape or weight in evaluating self during previous 6 months.

b Number of average episodes during the previous 3 months.

c Number of purging episodes (including vomiting, laxatives, diuretics, diet pills, and compensatory exercise) during the previous month.

d *df* = 149.

e *df* = 153.

**Table 3:** Comparison of lifetime psychiatric symptomatology between binge-first (BF) and diet-first (DF) groups as assessed by the SCID-IV

Category	Present	Absent	χ^2^	*p*	Φ
Any mood disorder					
BF	89 (73%)	33 (27%)	1.938	.164	.113
DF	18 (60%)	12 (40%)			
Any anxiety disorder					
BF	42 (35%)	78 (65%)	.029	.864	.014
DF	10 (33.3%)	20 (66.7%)			
Any substance disorder					
BF	46 (38%)	75 (62%)	9.963	.002	.257
DF	21 (70%)	9 (30%)			
Any Axis I disorder					
BF	102 (87.2%)	15 (12.8%)	0.884	.347	.078
DF	28 (93.3%)	2 (6.7%)			

Note: SCID-II = Structured Clinical Interview for DSM-IV.

### Risk Factors

No significant differences between BF and DF were identified with respect to risk factor domains (all ps > .01). However, an exploratory item-by-item analysis revealed that the DF group endorsed repeated sexual abuse (χ^2^_1_ = 9.14, *p* = .003, Φ = .24) before their index age significantly more than the BF group.

No significant differences were found in the overall number of antecedent life events experienced by either group (p > .01). Analyses comparing the two groups on specific antecedent events occurring in the year preceding their index age revealed that the BF group was more likely to report that someone close to them had died (χ^2^_1_ = 8.61, *p* = .003, Φ = .24). As derived from the PBI, there were no differences between the two groups on parental characteristics (all *ps* >. 01).

## Conclusion

The main purposes of the current study were to examine differences in risk factors and replicate previous findings on distinctive profiles of psycho-pathology between BF and DF subtypes among women with BED recruited from the community. Overall, no significant differences in the overall amount of risk factors emerged between the BF and DF groups, suggesting that a BF or DF onset of binge eating may not indicate distinct etiologic pathways in the development of BED. Although consistent with aforementioned etiologic theories, findings from exploratory item-by-item analyses only modestly add to the interpretation of overall negative findings. For example, the fact that women in the BF group were more likely to have someone close to them die in the year before the onset of their symptoms conforms to an interpersonal theory positing that an inability to regulate affective experiences, particularly when exposed to stress, contributes to the etiology of BED.^25^ The finding that the DF group experienced more sexual abuse is consistent with findings that a history of sexual abuse significantly increases the risk for the use of weight control techniques among adolescent girls, which would also be consistent with restraint theory.^38^,^39^ Methodologically, it is possible that the retrospective nature of this study was unable to detect risk factors at different stages of their development, timing that could adequately be assessed through a longitudinal design. Also of note for interpretation, power was sufficient for detecting group differences of medium size, but smaller effects may not have been found due to the small sample size of the DF group (see effect sizes).

Although no differences in risk factors emerged, the current study replicated some previous findings on distinctive profiles of the psychopathology of the BF versus DF subtyping. As expected, the current study’s BF group had a significantly earlier age of onset of both BED and binge eating than the DF group.^10^,^13^ However, in contradistinction to previous studies suggesting nearly equal rates of BF versus DF subtypes, the current study found a pre-ponderance of BF (81%) versus DF (19%) women. Also, unlike previous results that found the BF group endorsed more eating disorder and general psychopathology both in their history and at the assessment time,^9^,^10^,^13^,^40^ the current study found greater current symptomatology in the DF group, both in eating disorder psychopathology (Restraint and Weight Concern) and in history of psychiatric symptomatology (i.e., lifetime substance abuse). It is likely that women in the DF group had been more avid dieters throughout their lifetime. This constant experience of dieting may coincide with strong current eating disorder psychopathology. That the DF group endorsed a greater history of psychopathology is not surprising given the association seen in other samples between dieting and alcohol use (e.g., Krahn et al.^41^ and French et al.^42^).

The inconsistencies with previous research may have methodologic reasons, that is, the utilization of an interview-based assessment designed to minimize retrospective recall biases, sample characteristics, and/or the use of more stringent definitions of dieting and binge eating in determining the BF or DF subtype. In the current study, dieting was defined as attempts to eat less than most people (using <1200 kcal/day as a guide) for a sustained period (i.e., at least 3 months). This is a higher threshold of dieting than previous studies that used age of first weight-loss diet^10^,^13^,^43^; age when first lost ≥4.5 kg by dieting^11^,^15^,^44^; age when food restriction began^9^; or, ‘‘Were you on a ‘strict diet’ at the time binge eating began?’’^45^ The higher thresh-old of dieting in the current study may have led the DF group to exhibit more similarities to a BN population with higher eating psychopathology than to DF groups in previous studies.

Also, sample characteristics may have contributed to inconsistencies with previous findings. The sample used in the current study was a community-based and younger sample (upper age limit of 40 years) than previously studied samples that were older and treatment seeking. A treatment-seeking population may suffer longer with their disorder and/or suffer more severe symptoms that lead to treatment seeking than a community-based sample. Although the ages of onset of binge eating and BED between the BF and DF subtypes significantly differed in this study, this difference of approximately 5 years was less than the approximate 10-year difference in previous studies, which could also account for the lack of prominent differences between them.

From the results, it is evident that the descriptive profile and etiology of BED remain in need of further study. Using longitudinal research to assess when risk factors exert their influence in development, that is, their timing and patterning, could possibly aid in detecting differences.^46^ Other sub-types for BED, including dietary/dietary-depressive subtypes, have also been proposed. Additional investigation of different etiologic pathways would aid in elucidating the utility of subtyping, establishing the course for possible tailoring of treatment and preventive efforts.^8^ By building on the strengths of the current study (e.g., community sample, stringent definition of dieting and binge eating) and avoiding its limitations (e.g., sample size limitation), future research has the potential to specify risk factors and pathways leading to the development of BED.

## Footnotes

a The number of binge eating days was considered as the number of days in the previous month in which at least one objective bulimic episode (OBE) occurred, defined as the consumption of an unusually large amount of food, given the circumstances, accompanied by a sense of loss of control over eatingThe number of binge eating days was considered as the number of days in the previous month in which at least one objective bulimic episode (OBE) occurred, defined as the consumption of an unusually large amount of food, given the circumstances, accompanied by a sense of loss of control over eatingThe number of binge eating days was considered as the number of days in the previous month in which at least one objective bulimic episode (OBE) occurred, defined as the consumption of an unusually large amount of food, given the circumstances, accompanied by a sense of loss of control over eatingThe number of binge eating days was considered as the number of days in the previous month in which at least one objective bulimic episode (OBE) occurred, defined as the consumption of an unusually large amount of food, given the circumstances, accompanied by a sense of loss of control over eating^27^

b The six broad conceptually related risk domains (underlined), subdomains (in italics), and RFI individual items comprising each subdomain cover the following areas. Subject's Mental Health (*conduct problems:* conduct problems, truancy; *negative affectivity*: negative self-esteem, shyness, absence of friends, school anxiety, major depression; *substance abuse*: drug abuse, alcohol abuse; *perfectionism*: perfectionism, extreme compliance). Subject's Physical Health (*pregnancy history*: pregnancy, abortion, number of children; *severe childhood obesity*: obesity, advised to lose weight, diet prescribed). Other Environmental Experiences (*exposure to ethnic diversity*: neighborhood diversity, school diversity, diverse friends; *disruptions and deprivation*: food deprivation, frequent moves, change of caregiver). Family Weight and Eating Concerns (*family dieting*: number of dieting family members, dieting mother, dieting father, dieting sister; *maternal overweight*: mother’s highest weight, mother’s lowest weight; *family history of AN*: parental AN, number of family members with low weight, number of family members with AN; *paternal overweight*: father’s highest weight, father’s lowest weight; *family history of BN*: parental BN, number of family members with weight concern, number of family members with BN; *family overeating*: number of family members with objective overeating, number of family members with BED). Quality of Parenting (*maternal problem parenting:* maternal low care, maternal overprotection, maternal affectionless control; *family discord*: low contact with parent, parental arguments, parental low affection, family tension at mealtimes; *paternal problem parenting*: paternal low care, paternal overprotection, paternal affectionless control; *separations from parent*: separation from parent, boarding school; *parental absence or death*: parental absence from family, parental death; *high parental demands*: parental high expectations, parental criticism, and parental ill health). Parental Psychopathology (*parental mood and substance disorder*: parental major depression, parental alcohol problem, and parental drug problem).
